# AVIA: an interactive web-server for annotation, visualization and impact analysis of genomic variations

**DOI:** 10.1186/1753-6561-6-S6-P37

**Published:** 2012-10-01

**Authors:** Hue Vuong, Robert M Stephens, Natalia Volfovsky

**Affiliations:** 1 Advanced Biomedical Computing Center (ABCC), Information Systems Program, SAIC-Frederick Inc., Frederick National Laboratory for Cancer Research, Frederick, MD 21702, USA

## Background

A plethora of information that emerges from large-scale genome characterization studies has triggered the development of computational frameworks and tools for efficient analysis, interpretation and visualization of genomic data. Functional annotation of genomic variations and the ability to visualize the data in the context of whole genome and/or multiple genomes has remained a challenging task. We have developed an interactive web-based tool, AVIA [http://avia.abcc.ncifcrf.gov], to explore and interpret large sets of genomic variations (single nucleotide variations and insertion/deletions) to help guide and summarize genomic experiments.

## Material and methods

Our tool is based on coupling a comprehensive annotation pipeline with a flexible visualization method. We leveraged the ANNOVAR [1] framework for assigning functional impact to genomic variations by extending its list of reference annotation databases (RefSeq, UCSC, SIFT, PolyPhen, et cetera) with additional in-house developed sources (Non-B DB, PolyBrowse). Further, because many users also have their own annotation sources, we have added the ability to supply their own files as well. The results can be obtained in tabular format or as tracks in whole genome circular views generated by the Circos application [2]. Users can select different sets of pre-computed tracks, including whole genome distributions of different genomic features (genes, exons, repeats), as well as variations analysis tracks for the 69 CGI public genomes for reference.

## Results and conclusions

This version of AVIA is focused on gene-related impact assessment. Tracks showing the distribution of genes with variations of specific functional effects, such as non-synonymous variations, frame shifts, variable miRNA target sites or variations in G-quadruplexes in 5' UTRs, can be produced. Users can screen all RefSeq genes or other publicly available datasets or provide their own list of genes. Another option of the interactive analysis with AVIA is the ability to compare two input genomes whereby we can generate views of shared or distinct variations. This allows for comparison of tumor/ normal data sets to illustrate tumor-specific mutations. By exploring their work with AVIA, users can browse different tracks with their data and then re-generate signature plots to summarize the project.
